# Non-suicidal self-injury and impulsivity: Study of inibithory control in adolescent population

**DOI:** 10.1192/j.eurpsy.2021.545

**Published:** 2021-08-13

**Authors:** S. Pacifici, V. Baglioni, L. Cammisa, D. Guerrini, C. Mancini, G. Mirabella, A. Terrinoni

**Affiliations:** 1 Department Of Human Neuroscience, Sapienza University of Rome, Section of Child and Adolescent Neuropsychiatry, RM, Italy; 2 Department Of Clinical And Experimental Sciences, University of Brescia, Brescia, Italy

**Keywords:** NSSI, adolescent, impulsivity, inhibitory control

## Abstract

**Introduction:**

Non-suicidal self-injury (NSSI) is a clinical condition defined as intentional, self-inflicted act causing pain or superficial damage without suicidal intents (12-35% of the adolescent community). Several findings show a high correlation between NSSI and impairments in the impulsivity control.

**Objectives:**

The goal of our study is to evaluate the role of impulsivity in NSSI adolescents, relatively to the inhibitory control, in order to investigate if it can represent a neurocognitive risk factor underlying maladaptive behaviours and which psychopathological dimensions can be associated with this neurobiological process.

**Methods:**

30 NNSI inpatients (age range: 12 to 18 years), drug-free, were compared with an age-matched control group, using two behavioural paradigms for the study of inhibitory control: the Stop Signal task and the emotive go/Nogo. Psychopathological traits were evaluated by self-report questionnaires for impulsivity dimensions, suicidality and self-injurious acts. Statistical analyses were performed with SPSS program (p =0.05).

**Results:**

NSSI patients did not present impairments in the global inhibitory control but they had longer movement times in both paradigms and faster reaction times in the Go/no-go behavioural paradigm. Therefore, NSSI patients tended to be impulsive at an early stage of movement (rapid TR) and have to slow down in a second phase (TM slow) in order to have time to rework the cognitive processes underlying movement.

**Conclusions:**

The impulsivity dimension is a complex construct that involves multiple interconnected factors. The study of neuro-cognitive and psychopathological aspects and how they are interconnected is necessary to draw new perspectives on the etiopathogenesis of NNSI.

